# On the Development of a Cutaneous Flavonoid Delivery System: Advances and Limitations

**DOI:** 10.3390/antiox10091376

**Published:** 2021-08-28

**Authors:** Raquel Costa, Sofia A. Costa Lima, Paula Gameiro, Salette Reis

**Affiliations:** 1 LAQV, REQUIMTE, Departamento de Ciências Químicas, Faculdade de Farmácia, Universidade do Porto, 4050-313 Porto, Portugal; up201811987@fc.up.pt (R.C.); slima@ff.up.pt (S.A.C.L.); 2 LAQV, REQUIMTE, Departamento de Química e Bioquímica, Faculdade de Ciências, Universidade do Porto, 4169-007 Porto, Portugal; agsantos@fc.up.pt

**Keywords:** antioxidant, inflammation, skin research, topical delivery, transdermal delivery

## Abstract

Flavonoids are one of the vital classes of natural polyphenolic compounds abundantly found in plants. Due to their wide range of therapeutic properties, which include antioxidant, anti-inflammatory, photoprotective, and depigmentation effects, flavonoids have been demonstrated to be promising agents in the treatment of several skin disorders. However, their lipophilic nature and poor water solubility invariably lead to limited oral bioavailability. In addition, they are rapidly degraded and metabolized in the human body, hindering their potential contribution to the prevention and treatment of many disorders. Thus, to overcome these challenges, several cutaneous delivery systems have been extensively studied. Topical drug delivery besides offering an alternative administration route also ensures a sustained release of the active compound at the desired site of action. Incorporation into lipid or polymer-based nanoparticles appears to be a highly effective approach for cutaneous delivery of flavonoids with good encapsulation potential and reduced toxicity. This review focuses on currently available formulations used to administer either topically or systemically different classes of flavonoids in the skin, highlighting their potential application as therapeutic and preventive agents.

## 1. Introduction

**Figure 1:**
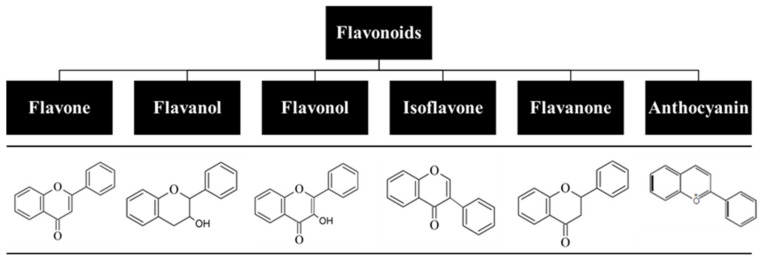
Classification of flavonoids based on their chemical structure.

For centuries, flavonoids have been used to treat various human diseases, and despite the fast-growing development of new and innovative synthetic drugs, continuous use of these natural compounds has prevailed to this day [1,2]. Flavonoids are one of the key classes of bioactive compounds abundantly found in plants and have a general structure of a 15-carbon backbone, consisting of two benzene rings connected by a 3-carbon bridge, which forms a heterocycle. They are low-molecular-weight polyphenolic compounds derived from plant metabolites, and the presence of different substitutes creates different subclasses (Figure 1) [3,4,5]. Due to their broad spectrum of biological activity and attractive pharmacological properties, which include antioxidant, anti-inflammatory, antiproliferative, photoprotective, and antiaging effects, flavonoids have been explored as a therapeutic option towards a great number of diseases, including several skin disorders [6,7]. However, their lipophilic nature, which results in a reduced capacity to be orally absorbed, and the fact that they undergo extensive first-pass metabolism and rapid elimination hamper their oral bioavailability [8,9,10]. Thus, alternative research focuses on the development of the cutaneous delivery of flavonoids, with high patient compliance and potential to surpass drawbacks associated with oral and parental routes of administration. Although skin acts as a physical barrier to drug absorption, the development of delivery systems, such as nanoparticles, hydrogels, and microneedles, allows for the delivery of both hydrophilic and lipophilic compounds as well as drugs with shorter half-time and limited therapeutic index. This results in a higher bioavailability at the target site under a controlled release rate and avoids interactions with gastric and intestinal fluids as well as flavonoid degradation [11,12].

This review focuses on the therapeutic potential of flavonoids, including their mechanisms of action and influence of several delivery systems for topical application on the improvement of their bioavailability, safety, and therapeutic capacity. In addition, current in vitro and in vivo studies of different classes of flavonoids under study for its application on the treatment of skin conditions are highlighted.

## 2. Human Skin: Structure and Function

The skin is the largest organ of the body and acts as a physical barrier that separates the body from the external environment. It constitutes a first line of defense in protecting the body against physical, chemical, and microbial insults and assists in a wide range of functions such as prevention of body’s dehydration, thermoregulation, sensation, and synthesis of vitamin D.

**Figure 2:**
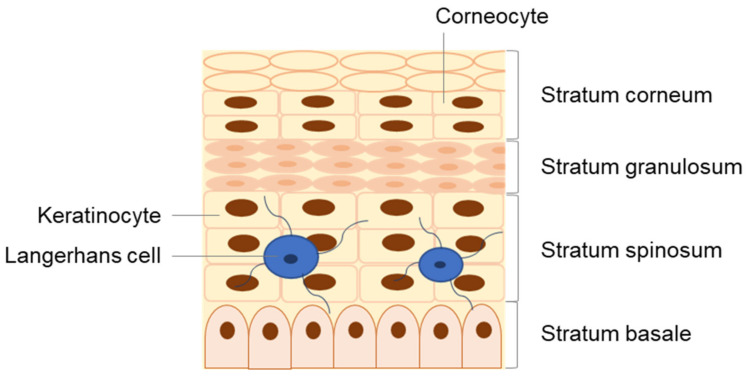
Detailed structure of the epidermis, composed of four distinct strata: the *stratum basale*, *stratum spinosum*, *stratum granulosum*, and *stratum corneum*.

The skin is divided into three major layers, namely the epidermis, dermis, and hypodermis [13,14,15,16]. The epidermis is the outermost viable layer of the skin and constitutes a barrier between the body and the external environment. As represented in Figure 2, the epidermis is composed of four layers: the *stratum basale*, *stratum spinosum*, *stratum granulosum*, and *stratum corneum* (*SC*). An additional layer, the *stratum lucidum*, which is often considered the lower part of the *stratum corneum* as opposed to an individual epidermal layer, can be found on the palm and sole of the foot, parts of the body with thickened skin. In addition, appendageal features such as hair follicles and sweat ducts are transversal to multiple skin layers [14].

The dermis, with a thickness of typically 1–2 mm, comprises the bulk layer of the skin and provides its elasticity, flexibility, and tensile strength. It is composed of collagenous and elastin fibbers, which accommodate epidermally derived appendages such as hair follicles, nails, sebaceous glands, and sweat glands as well as sensory nerve endings, lymphatic vessels and blood capillaries, which extend to the dermal side of the dermo-epidermal junction, thus allowing for metabolic exchanges and waste removal between the epidermis and the blood system [15]. The dermis contains resident cells, primarily fibroblasts that synthesize type I collagen for the extracellular matrix, as well as cells from the immune system, including macrophages and dermal dendritic cells (DCs). Below this layer, the fibrous connective tissue starts to transition to the adipose tissue of the hypodermis, where adipocytes interconnect with the collagen fibers, forming a thermal barrier for energy storage and protection from physical shock [15,17].

The hypodermis is the innermost layer of the skin and may be considered part of the endocrine system. It provides the nerves, and the lymphatic and blood vessels, which permeate into the upper layers, thus playing a critical role in re-epithelization, wound healing, and angiogenesis [14,18].

## 3. The Skin as an Immune Organ

The skin is undoubtably a complex organ that harbors a highly specialized immune microenvironment essential for maintaining tissue homeostasis, defense, and repair. Through a sophisticated network of resident immune and non-immune cells, biomolecules, and skin structures, the skin is able to protect the host from pathogen invasion as well as chemical and physical stress [13,14,15].

Resident immune cells (e.g., melanocytes and Langerhans cells) ensure tissue function in homeostasis and actively seek environmental antigens. Following an infection or tissue injury, these cells create a defense network in order to fight the insult and to restore the tissue to its original state [19,20]. Both epidermal keratinocytes and Langerhans cells (LCs) as well as dermal DCs, mast cells, and macrophages function as sentinels that not only provide a protective barrier but also trigger an early response to pathogen invasion by releasing stored antimicrobial peptides (AMPs), chemotactic proteins, and cytokines [20].

### 3.1. Non-Immune Cells as Key Immunological Mediators

Keratinocytes in response to multiple stimuli produce large amounts of interleukins (ILs), tumor-necrosis factor (TNF), and antimicrobial peptides, which trigger local immune responses. Moreover, they produce chemokines and immunoregulatory cytokines that act on resident immune cells such as DCs, mast cells, and macrophages, triggering the upregulation of inducible mediator expression and the recruitment of additional immune cells to the site of inflammation [21].

Similar to keratinocytes, fibroblasts also exert key immunomodulatory features. They express pattern recognition receptors (PRRs), produce AMPs, and synthesize many cytokines.

### 3.2. Immune Skin Cells

Langerhans cells are the only myeloid cell type in the epidermis. These cells act as key immunological mediators, with both an antigen-presenting role and a possible tolerance induction during an infection. These cells take up and process microbial fragments and lipid antigens and present them to effector T cells [19]. LCs are naturally migratory cells that continuously search the skin for signs of infection and that drain lymph nodes in order to build tolerance in homeostasis or to initiate adaptive immune responses. In addition, they can further exert immunoregulatory and tolerogenic functions [22,23,24].

Mast cells are commonly found in the upper dermal layer of the skin, actively protecting it and responding to infections, venoms, and stress caused by wound healing [20]. Mast cells produce and release significant amounts of histamine, thus being naturally involved in allergic reactions, and are recognized as typical allergy cells. Recent studies show their critical role in wound healing, inflammation, angiogenesis, immune tolerance, and cancer [19].

Dermal DCs, similar to LCs, are prime antigen-presenting cells, the main role of which is to provide immunosurveillance against pathogens. These cells activate and promote the clonal expansion of skin-resident memory CD4^+^ or CD8^+^ T cells. T cell-derived pro-inflammatory cytokines and chemokines can in turn stimulate epithelial and mesenchymal cells, therefore intensifying the inflammatory response [25]. Plasmacytoid DCs are a type of DC found in the skin exclusively during an inflammatory stage. These cells produce large quantities of interferon-α (IFN-α), essential for viral defense. In addition, they have also been implicated in autoimmune disease such as psoriasis as well as fibrosis [26].

Table 1 summarizes the functions of the main cell types found in the skin and their role in the skin immunology, which leads the outcome of molecules delivered cutaneously.

## 4. The Skin as a Barrier in Cutaneous Delivery

Cutaneous delivery is one of the most attractive routes of administration for drugs and cosmetics, since it can overcome the many drawbacks of most common routes (e.g., parenteral and oral), including low bioavailability and cytotoxicity, while ensuring a sustained drug release at the desired site of action [32]. However, normal skin presents a serious barrier to drug absorption, mostly due to the unique lipid composition and organization of the SC, which plays a key role in skin permeability and therefore drug permeation through the skin [32,33,34].

Despite recent advances in the identification and elucidation of the mechanisms of drug transport through the skin and the generation of structure–activity relations that allow for an accurate prediction of the permeation profile of a drug, the development of new formulations and drug delivery systems capable of improving drug uptake via the skin barrier are still needed [5]. This is particularly relevant when it comes to routes for flavonoid administration. It is now well-established that, due to its lipophilic nature, the cutaneous route is the best delivery approach for flavonoids. In fact, an array of novel formulations for topical delivery have been developed and optimized in order to increase the solubility and permeability of flavonoids across the skin barrier [5]. Nonetheless, there are still major challenges to overcome in order to successfully deliver these compounds to the skin for therapeutic purposes, including inadequate residence time and sustained release profile as well as the scalability of formulation and manufacturing process [1,3,4,5].

Targeting the optimal skin penetration pathway is an essential step for effective topical drug delivery. On that matter, drugs can be administrated through the skin in an invasive and noninvasive way. In the invasive route of administration, drugs can permeate through the skin via needle injections (subcutaneous, intramuscular, or intravenous routes) or via the implantation of a device [35]. In the subcutaneous route, the needle is inserted directly into the fatty tissue, thus reaching the bloodstream. For instance, insulin, similar to other proteins that are destroyed in the digestive tract, is administrated through this route. For larger volumes of drugs, the intramuscular route is preferred in comparison with the subcutaneous one. On the other hand, in the intravenous route, the drug is delivered directly into the bloodstream, in a well-controlled and rapid manner. The implantation of a device inserted under the skin is another invasive drug administration method and is usually considered when a controlled release of the drug with time is needed.

**Figure 3:**
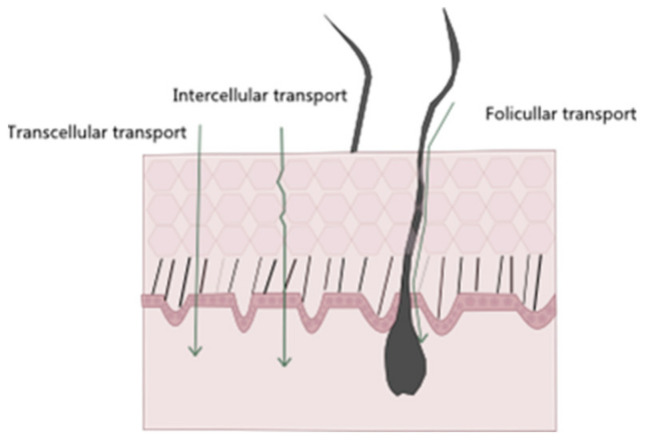
Schematic representation of different entry pathways for molecules into the skin.

Regarding noninvasive drug administration methods, there are four possible pathways of drug permeation across the skin: the intracellular, intrafollicular, transcellular, and polar pathways (Figure 3) [36]. The intrafollicular route, sometimes classified as the appendageal route, encompasses drug permeation through the skin appendages, such as lipophilic follicular ducts, sebaceous glands, or hydrophilic sweat ducts [14,37]. In the most commonly used pathway, the intercellular one, the drug travels through the lipid matrix that occupies the intercellular spaces between the corneocytes, thus making it the preferred permeation route for lipophilic molecules. On the other hand, in the transcellular way, also known as the intracellular pathway, the drug diffuses through the various skin layers and dead cells, allowing for the transport of hydrophilic or polar molecules. Finally, in the polar pathway, the drugs permeate through the skin via polar pores available at its surface. This observed flux of drugs across the various layers of the skin is called transdermal drug delivery [15,18,38,39].

After passing through the SC and diffusing through the viable epidermis and dermis, the drug becomes available for its uptake into the systemic circulation [5]. Systemic absorption depends on the application site, its area, and the nature of the delivery system. Another alternative to the oral administration of drugs is topical delivery, in which the drug is intended to be absorbed at specific areas of the skin rather than being targeted for systemic delivery. Examples of drugs topically delivered to the skin include corticosteroids, antifungals, antivirals, antibiotics, antiseptics, and local anesthetics [40].

## 5. Flavonoids: Relevant Biochemical and Biological Properties

In addition to their well-reported strong antioxidant activity, flavonoids also exhibit the ability to modulate key cellular signaling pathways and enzymatic reactions involved in a wide range of pathophysiological events such as cell proliferation, inflammation, immune response, platelet aggregation, and cytotoxicity [41,42,43,44,45]. Studies indicate that the biological properties of flavonoids are beneficial in solving or controlling skin disorders. The following subsections briefly describe the antioxidant, anti-inflammatory, anticancer, and antibacterial activities of flavonoids, elucidating the molecular targets and mechanism of actions with an effect on skin disorders (Table 2).

### 5.1. Antioxidant Properties

One of the best-described properties of flavonoids is their capacity to act as powerful antioxidants. In fact, flavonoids have the ability to act as free-radical scavengers and metal ion chelators as well as the capacity to affect enzymatic and non-enzymatic systems that regulate cellular redox balance [41,45,53]. Their mechanisms of antioxidant action can include (1) the suppression of reactive oxygen species formation (ROS) either through inhibition of certain enzymes or by chelating trace elements involved in the generation of free radicals, (2) scavenging ROS, and (3) the upregulation or protection of antioxidant defenses [3].

Depending on their structure, there is considerable evidence that flavonoids are effective scavengers of ROS, such as peroxyl, alkyl peroxyl, hydroxyl, and superoxide radicals as well as reactive nitrogen species (RNS), in particular, nitric oxide (NO) and peroxynitrite (ONOO^−^). Due to the presence of vicinal hydroxyl groups, several flavonoids can also act as chelators of redox-active metal ions, such as copper and iron, thus preventing free radical formation and lipid peroxidation [54,55,56,57].

Free metal ions enhance the formation of ROS through the reduction of hydrogen peroxide to the highly reactive hydroxyl radical. Flavonoids, due to their lower redox potential are able to reduce highly oxidizing free radicals such as hydroxyl, superoxide, and peroxyl radicals by donating a hydrogen atom. The presence of a 3′,4′-catechol group in the flavonoid structure, for example, is known to enhance their capacity to inhibit lipid peroxidation. This trait makes flavonoids highly effective scavengers of peroxyl, superoxide, and peroxynitrite radicals [58,59,60,61]. Epicatechin and rutin, for instance, were shown to be strong radical scavengers and inhibitors of lipid peroxidation in vitro [3]. Moreover, they are known to inhibit enzymes involved in ROS formation, such as the case of xanthine oxidase, myeloperoxidase, and NADPH oxidase.

On the other hand, flavonoids have the capacity to upregulate both enzymatic and non-enzymatic systems involved in the removal and detoxification of oxidant species, particularly reduced glutathione (GSH), GSH peroxidase, GSH reductase, GSH S-transferase, superoxide dismutase, and catalase, as it has been demonstrated in animal models for rutin; quercetin; daidzein; and to a lesser extent, genistein [62,63]. Certain flavonoids, such as quercetin and the catechins, have been shown to regenerate ascorbate and α-tocopherol via electron transfer reactions, thus displaying an additional antioxidant mechanism [64,65,66].

It is noteworthy that, under certain conditions, flavonoids might also exert a marked pro-oxidant activity, becoming cytotoxic. In fact, they can undergo transition metal or peroxidase-catalyzed reactions, which lead to the formation of highly reactive oxygen species that can damage proteins and DNA [67].

### 5.2. Anti-Inflammatory Properties

Inflammation is a biological response to tissue injury, microbial infection, and chemical irritation. During an inflammatory process, the migration of immune cells from blood vessels and the release of mediators to the site of damage are followed by the recruitment of inflammatory cells and the release of ROS and pro-inflammatory cytokines that work together to eliminate pathogens and to repair injured tissues. Flavonoids are known to significantly affect the immune system [47,68]. For instance, hesperidin, apigenin, and quercetin are among a broad spectrum of flavonoids known for their anti-inflammatory and analgesic capacity. In fact, studies have shown that, both in vitro and in vivo, flavonoids have the capacity to downregulate the expression of a wide range of pro-inflammatory genes, including the inducible nitric oxide synthase (iNOS), cyclooxygenase (COX), lipoxygenase (LOX), and several key cytokines, mainly through the inhibition of the mitogen-activated protein kinase (MAPK)- and nuclear factor-kappa B (NF-κB)-mediated signaling pathways [68,69,70]. This ability to inhibit the arachidonic acid pathway at the level of phospholipase A_2_, COX and LOX is of particular importance since it results in a decrease in the production of prostaglandins and leukotrienes, essential mediators of the acute inflammatory response [71,72]. Flavonoids are in fact known to modulate several steps of the inflammatory cascade both in human and animal cell types. Quercetin, kaempferol, genistein, and epigallocatechin-3-gallate (EGCG) are among the flavonoids that have been extensively studied on their ability to affect iNOS activity and NO production. They have been found to inhibit iNOS expression via the downregulation of extracellular signal regulated protein kinase 1/2 (ERK 1/2) and p38 MAPK phosphorylation and by preventing the binding of NF-κB to the iNOS gene promoter [71,73,74,75,76,77]. In addition, several flavonoids have been shown to interfere with the production and function of various pro-inflammatory cytokines, chemokines, and adhesion molecules, such as TNF-α; IL-1β, -6, and -8; monocyte chemotactic protein-1 (MCP-1); macrophage inflammatory protein-2 (MIP-2); vascular cell adhesion molecule (VCAM); and P-selectin by inhibiting the MAPK pathways, by blocking NF-κB nuclear translocation, and via COX-2 synthesis [78,79,80,81,82,83,84].

### 5.3. Anticancer Properties

Recent studies have demonstrated a direct link between flavonoids and their ability to prevent the development of different types of malignant tumors both in human and animal models. In fact, it is now well-established that flavonoids exert an anticarcinogenic effect by quenching oxidative stress and inflammatory response; by inducing apoptosis; by suppressing MMP secretion; and by inhibiting cell growth, tumor cell invasion, and angiogenesis [85,86,87,88,89,90,91].

Flavonoids might act on the initial steps of cancer development by preventing the DNA damage that can be induced by free radicals and carcinogens. In addition, flavonoids have been demonstrated to inhibit various types of cancer cell proliferation by inducing cell cycle arrest at the G1/S or G2/M phases through the downregulation of cyclins and cyclin-dependent kinases. They were also shown to stimulate apoptosis through the activation of caspases 3, 9, and 8 and proapoptotic proteins p53, p27, and Bax as well as via the inhibition of antiapoptotic proteins (Bcl-2 and Bid) and the release of cytochrome c [92,93,94,95,96,97,98].

Their antiproliferative and proapoptotic activity might implicate the inhibition of growth factors and its receptors such as platelet-derived growth factor (PDGF), PDGF receptor (PDGFR), and epidermal growth factor receptor (EGFR) in addition to the activation of NF-κB and the inhibition of the Akt/PI3K, ERK and activating protein-1 (AP-1) pathways [99,100]. For instance, flavonoids such as EGCG, quercetin, genistein, luteolin, and the anthocyanins were able to reduce angiogenesis, a key event in tumor growth, invasion, and metastasis via the downregulation of VEGF, VEGFR, PDGF, PDGFR, EGFR, and MMP. Other flavonoids were also shown to affect cancer cell adhesion and movement by inducing cytoskeletal modifications, by inhibiting cell adhesion to fibronectin, by reducing integrin expression and disrupting the stress fibers, and by reducing myosin II regulatory light chain phosphorylation [86,87,88,101,102,103].

### 5.4. Antibacterial Properties

Flavonoids are naturally synthesized by plants in response to microbial infection. Similarly, it has been found that they exert in vitro antimicrobial activity against a wide range of microorganisms. In fact, flavonoids such as apigenin, galangin, flavonol glycosides, isoflavones, and flavanones have all been shown to possess strong antibacterial activity [1]. Given their antibacterial properties, flavonoids are being used as wound healing agents.

## 6. Bioavailability of Flavonoids

One of the major concerns regarding the use of flavonoids as therapeutic agents is their relatively low bioavailability. Even in the presence of a large daily intake of flavonoids in dietary sources, their plasma and tissue concentrations are often insufficient to exert the desired pharmacological effects [3]. Due to several factors that include chemical structure and molecular weight, relatively low water solubility, absorption and metabolism in the gastrointestinal tract, lack of site specificity in distribution, and rapid elimination, flavonoids have generally low bioavailability, which largely affects their therapeutic potential. Moreover, this class of compounds is highly susceptive to degradation upon oxygen exposure, temperature changes, ultraviolet radiation, or pH change [104,105,106].

After being absorbed by the intestinal epithelium, flavonoids undergo extensive biotransformation into conjugated products, namely glucuronides, sulphates, and methylated derivatives, first in the intestine and then in the liver, where they are secreted into bile [107,108]. Thus, the bioavailability and the subsequent cell and tissue accumulation of the different flavonoids essentially depend on the multidrug-resistance-associated proteins (MRP-1 and MRP-2), ubiquitously expressed as ATP-dependent efflux transporters. The actual flux of a flavonoid from the gut lumen to the blood stream and the various organs depends on the tissue distribution of MRP-1 and MRP-2 as well as on their substrate’s affinity. This metabolic pathway is called phase III metabolism. However, it appears that certain phase II metabolic derivates of flavonoids can act as competitive substrates of the MRP-mediated membrane transporters and the potential use of flavonoids as a mean to overcome transporter-mediated chemotherapy resistance due to the frequent overexpression of MRP in several types of cancer is based on this property. The intestinal absorption of quercetin, for instance, is favored in the aglycone form, and its metabolism in the gut and liver appears to be relatively high, so that less than 2% of ingested quercetin is recovered on the plasma [3]. Additionally, after oral administration of flavonoids, a significant amount can reach the colon and can interact with microbiota. Microbiota can, for instance, metabolize some flavonoids to smaller phenolic compounds with similar biological effects and improved bioavailability; however, on the other hand, it can also extensively metabolize flavonoids via the glucuronidase and sulfatase enzymes, cleaving the heterocycle break and producing inert polar compounds that are rapidly excreted without producing any biological effect [104].

In addition, flavonoids have been reported to significantly inhibit the activity of the cytochrome P450 system, which can result in an increase of the half-life and concentration of many drugs, thus enhancing their toxicity and side effects [109]. Flavonoids such as quercetin, ECG, EGCG, and sylibin have been shown to downregulate the cytochrome CYP3A4, which is the major cytochrome P450 isoenzyme in the intestine and is responsible for the metabolism of approximately 50% of all prescribed drugs, thus increasing the risk of potential toxicity, especially of drugs with a limited therapeutic window [110]. Flavonoids can also interact with ATP-binding cassette (ABC) transporters, inhibiting them, which can increase the bioavailability of poorly available drugs, on the one hand, but it can also potentiate the toxicity of other ABC transporters substrates [111]. Thus, flavonoid encapsulation in effective nano-carrier systems can not only improve their pharmacokinetics and therapeutic potential but also avoid enhancement of the toxicity and side effects of drugs that can concomitantly be administrated with these compounds [104].

The rapid metabolic elimination of flavonoids, together with the evidence that they are able to interact with the metabolism of other drugs, highlights the need to develop novel ways to improve the delivery of flavonoids. Cutaneous administration emerges as an alternative option to common oral and parenteral routes [112,113]. Skin drug delivery is one of the most preferred administration routes with higher patient compliance and satisfaction. The advantages also include the avoidance of liver first pass metabolism effects, metabolic degradation associated with oral administration, and minimal systemic side effects.

## 7. The Need for Nanocarriers in Cutaneous Flavonoid Delivery

Despite flavonoids’ pharmacological potential, dietary flavonoids present several disadvantages, mentioned in Section 6, hindering their clinical potential. In addition, the fact that flavonoids can suffer an enhanced complexation or precipitation when ingested with other food components as well as degradation by microbiota greatly reduces their bioavailability and stability. On that matter, cutaneous delivery is one of the most advantageous routes in overcoming the challenges associated with flavonoid administration [3,104].

**Figure 4:**
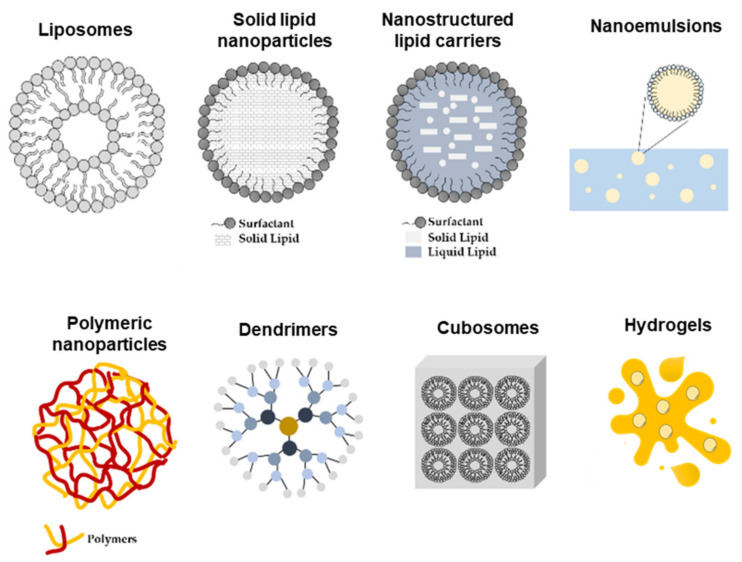
Schematic representation of nano-delivery systems used for topical skin delivery.

Nonetheless, the impermeable nature of the skin presents a serious challenge to cutaneous delivery, where in most of the cases the therapeutic effect produced by the conventional drug dosage is not sufficiently effective. Thus, the development of nano-engineered delivery systems for flavonoids capable of increasing the solubility and bioavailability and of providing a site-specific delivery with improved pharmacokinetic properties is imperative. Thus far, gels are the most common form of topical drug administration, including hydrogels and olegels. However, other delivery systems such as lipid and polymeric nanoparticles, microparticles, and transferosomes, among others, are currently being developed (Figure 4). These carriers can later be formulated into creams and gels, improving patient compliance [5].

### 7.1. Nano-Delivery Systems: Advantages and Limitations

The development of novel drug delivery systems, which allow for the cutaneous delivery of otherwise poorly effective compounds with undesirable physicochemical and pharmacokinetics parameters, can improve their efficacy and safety. Nanotechnology tools designed for skin drug delivery include microdevices (1–1000 µm) and nanodevices (1–1000 nm) for drug delivery [112]. Micro-delivery vehicles can act as reservoirs for a drug that is released into the tissue interstitial space. Due to their size, they can cross the skin barrier and directly deliver the drug to the site of action, minimizing toxicity and prolonging release [3,51].

Despite great progress, the development of a successful drug delivery system is still a challenging task that requires meticulous selection of the vehicle according to the active agent. In fact, the safety of the chosen materials, eventual harmful degradation products, and high cost of the final product are major limitations that need to be addressed.

The use of nanocarriers allows for an improvement in crucial drug properties, including solubility, diffusivity, blood circulation half-life, and immunogenicity. However, there are some essential prerequisites for the development of a successful targeted drug delivery vehicle, including the physicochemical and biological properties of the vehicle [114]. For instance, size, charge, and surface hydrophilicity are all properties that can impact the circulating half-life of the particles as well as their biodistribution. Small molecule-, peptide-, or nucleic acids-loaded nanoparticles are not as easily recognized by the immune system; in addition, the presence of targeting ligands can increase the interaction of drug delivery systems with the cells and can enhance cellular uptake by receptor-mediated endocytosis [115]. Nevertheless, there are some limitations on the use of nanocarriers, namely storage, generation of pro-oxidant chemical species, and unexpected pro-inflammatory response, which need to be considered in their design.

In summary, the advantages of nanocarriers application for cutaneous drug delivery include (1) targeted delivery, with maximized efficacy and minimized systemic side effects; (2) controlled drug release; (3) prolonged half-life in the systemic circulation; (4) improved patient compliance; (5) improved drug solubility and permeability; (6) protection against degradation; (7) delivery of multiple drugs with a synergistic effect; and (8) improved biocompatibility [3,115,116,117].

### 7.2. Nano-Delivery Systems Applied for Flavonoid Cutaneous Administration

Among the numerous nano-based drug delivery systems that have been developed so far, lipid-based nanoparticles, including liposomes and lipid nanoparticles as well as polymeric-based nanoparticles, are most commonly used for flavonoid delivery [3]. Liposomes are concentric vesicles consisting of an aqueous core surrounded by a membranous lipid bilayer that, thanks to their structure, can encapsulate hydrophilic, hydrophobic (in the lipid bilayers), and amphipathic molecules. To avoid the rapid elimination of liposomes from the blood by the cells of the reticuloendothelial system (RES), primarily in the liver and spleen, their structure can be modified by coating their surface with inert and biocompatible polymers such as polyethylene glycol (PEG) [118,119,120,121].

Solid lipid nanoparticles (SLN) are nanocarriers composed by a solid hydrophobic core and stabilized by a surfactant. Among the main advantages of using SLN as drug carriers, their high stability and capacity to protect the incorporated drugs from degradation, the controlled drug release, site-specific targeting, and good biocompatibility stand out. However, they often display low loading capacity as well as a short storage time with frequent drug expulsion. SLN can be administered by the parenteral, oral, transdermal, dermal, and ocular routes. In addition, they have higher stability compared with liposomes and, due to their easy biodegradability, are less toxic than polymeric nanoparticles, making them highly versatile drug delivery vehicles. Their primary applications target skin disorders; for example, curcumin loaded in SLNs featured a controlled drug release over 24 h and effective skin deposition for the reduction in pigmentation and inflammation in Balb/c mouse skin [117,122,123].

Regarding its potential application as a cutaneous drug delivery system, SLN-enhanced SC permeation is attributed to (1) prolonged contact with the skin surface; (2) their occlusive nature, since they form a film on the surface of the skin that combines with the skin lipids promoting a reduction in water loss and hydration of the skin; and (3) the interaction between the lipids in the nanoparticles and SC lipids, which facilitates permeation of lipid-soluble compounds.

The use of cationic lipids on the nanoparticle’s composition allows for an interaction with the negatively charged skin surface. For instance, a highly positively charged (+51 mV) SLN using cationic phospholipids, tween 20 as a surfactant, tricaprin as a solid lipid core, and encapsulating plasma DNA was shown to have enhanced in vitro permeation into mouse skin and the expression of mRNA in vivo after topical application [124].

Liquid lipids (oils) can be added to a solid lipid, creating an irregular lipid matrix, called the nanostructured lipid carriers (NLC). The lipids’ spatial structure allows for an increased drug loading capacity and better stability compared with SLN. Studies have shown that both NLC and SLN display similar mechanisms of skin permeation enhancement, through occlusion and mixing between the formulation and the SC lipids, although the presence of a liquid lipid is known to increase the solubilization and loading capacity, thus resulting in greater skin deposition [3,124].

Polymeric nanoparticles are colloidal structures composed of natural or synthetic polymers. Depending on their shape, they can be classified as nanocapsules, vesicular systems with the drug in a core surrounded by a polymeric membrane, and nanospheres, which are porous matrixes in which the drug is uniformly dispersed [125,126]. The most common synthetic polymers used in the preparation of these nanoparticles are poly(lactic acid) (PLA), poly(lactide-co-glycolide) (PLGA), poly(methyl methacrylate) (PMMA), and poly(alkylcyanoacrylate) (PACA) [127,128,129,130,131,132]. In addition, natural polymers such as alginate, gelatin, chitosan, and albumin are also frequently used since they are less toxic compared with synthetic polymers. Polymeric nanoparticles feature biocompatibility, biodegradability, stability, and surface modification potential, therefore allowing for the controlled release of both hydrophobic and hydrophilic compounds as well as proteins, peptides, or nucleotides to the specific site of action. To avoid rapid removal from blood and to reduce its cytotoxicity, polymeric nanoparticles can be covered with a non-ionic surfactant or coated with hydrophilic substances such as PEG or carbohydrates, thus reducing opsonization [3].

## 8. Cutaneous Delivery Systems of Flavonoids for Treatment of Skin Pathologies

**Figure 5:**
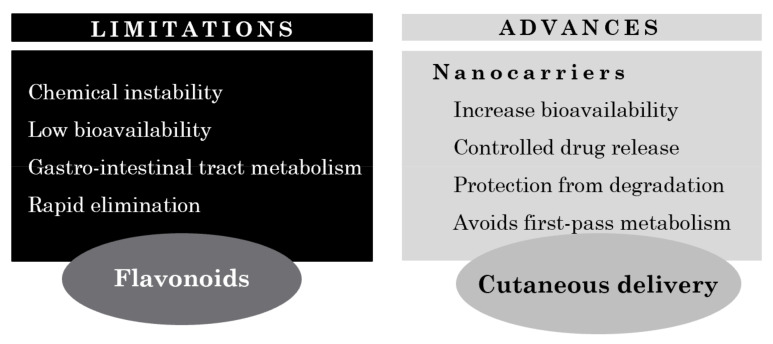
Limitations and advances on cutaneous flavonoid delivery.

Cutaneous delivery of flavonoids is a powerful strategy to avoid systemic toxicity while restricting the therapeutic effects to the specific site of action. However, one of the major challenges that a topical delivery system faces is the ability to overcome the *SC* barrier against foreign substances [5]. In addition, most flavonoids are highly lipophilic compounds and their permeation across the *SC* into viable skin layers is hindered by their affinity for SC components and the tendency to be retained in this layer. Thus, there has been a growing interest in the use of nanotechnology as a strategy for a more efficient flavonoid delivery to the human body (Figure 5). Nano-delivery systems are in fact excellent tools to overcome the challenges associated not only with the cutaneous absorption of the drug per se but also with flavonoid pharmacology, including low solubility, short half-life, and poor bioavailability [5,124].

### 8.1. Examples of Nanocarriers Designed for Flavonol Cutaneous Delivery

Flavonols are O-glycosidic ketonic compounds with a sugar moiety at the 3-position that act as powerful antioxidants, protecting the skin from ROS formation. Compounds belonging to this family of flavonoids are quercetin, kaempferol, and myricetin, among others [133].

Quercetin, one of the best studied and most common flavonoid found in nature, was shown to have poor permeability across excised human skin [4]. For that, this flavonoid has been incorporated into different delivery systems, including nanoemulsions, nanocapsules, lipid nanoparticles, and microemulsions, to increase its solubility and skin permeability [5]. Casagrande and colleagues incorporated quercetin into two different oil-in-water emulsions with a distinct lipid content in order to evaluate their potential application as a topical delivery system. The in vivo results demonstrated that these formulations were an effective vehicle for topical application of quercetin with the goal of controlling ultraviolet B (UVB)-induced skin damage [4,134]. Based on these results, other studies were conducted to design novel delivery systems to increase quercetin effectiveness when topically applied. For example, quercetin was incorporated into a liquid, crystalline formulation and the influence of this vehicle in the antioxidant activity of this flavonoid was evaluated in vitro. The presence of a liquid, crystalline structure allowed for an easier diffusion through the skin and a considerable solubilizing capacity for both oil- and water-soluble compounds. Scalia and colleagues also demonstrated that the incorporation of quercetin in lipid microparticles improved its photostability and chemical stability as well as its biocompatibility [135,136]. In another study, Tan and colleagues investigated the potential of using lecithin-chitosan nanoparticles as a topical delivery system for quercetin. Compared with quercetin in its free form, the quercetin-loaded nanoparticles displayed higher permeation ability and significant accumulation of quercetin in the skin, particularly in the epidermis. In addition, microstructure observations of the skin surface following administration showed that the interaction between constituents of the nanoparticles and the skin surface markedly changed the morphology of the *SC* and disrupted the corneocyte layers, therefore facilitating permeation and accumulation of quercetin in the skin [137].

Nan and colleagues evaluated the efficacy of topically applying quercetin-loaded chitosan nanoparticles against UVB radiation. The authors demonstrated that quercetin, if entrapped into chitosan nanoparticles, could be efficiently up taken by HaCaT cells (keratinocytes) and could easily permeate through the epidermis layer while displaying better stability and lower cytotoxicity. Moreover, they also found that quercetin-loaded nanoparticles could enhance the effects of this flavonoid when inhibiting the NF-kB/COX-2 signaling pathway as well as when ameliorating the skin edema caused by UVB radiation [138].

Bose and Michniak-Kohn developed a solvent-free NLC formulation of quercetin using probe ultrasonication and evaluated the feasibility for topical delivery. Formulation factors such as the nature of the lipid (solid/combination of solid and liquid) in the SLN and NLC systems and the drug loading capacity were evaluated to produce the optimal formulation with an adequate physical stability. Overall, the NLC system showed the highest improvement in the topical delivery of quercetin, manifested by the amount of quercetin retained in full-thickness human skin compared with a control formulation with a similar composition and particle size in the micrometer range, thus demonstrating the feasibility of NLC systems for improved cutaneous delivery of this compound [139].

Penetration enhancer containing vesicles (PEVs) are also known to be powerful enhancers for dermal delivery due to the presence of both phospholipids and penetration enhancers (PE), which provide a synergistic effect on skin permeation. PE increases the fluidity of the lipids of the SC, facilitating the delivery of drug-loaded vesicles and its subsequent diffusion through the skin [5]. Hence, in 2011, Chessa and colleagues developed quercetin-loaded PEVs, formulated using four different hydrophilic PE, and characterized them by size, surface charge, loading capacity, and morphological and viscoelastic features. In addition, their penetration capability and distribution through pig skin were assessed to obtain the optimal formulation for the delivery of quercetin to the skin [140]. In another study, performed by Caddeo and colleagues, quercetin-loaded phospholipid vesicles, in particular liposomes and PEVs, were developed in order to study their efficacy on 12-O-tetradecanoylphorbol-13-acetate (TPA)-induced skin inflammation. In vivo results demonstrated that the vesicles, in particular PEVs, were capable of delivering the drug to the inflammation site, that is the dermis, inhibiting oxidative stress and leukocyte accumulation as well as stimulating the repair of skin damaged induced by TPA. Through this work, the authors highlighted the use of vesicular systems, particularly PEVs, as a delivery vehicle for flavonoids, with a therapeutic potential to treat inflammatory skin disorders [141].

Kaempferol is another well-known flavonol with antioxidant, anti-inflammatory, anticancer, and antiallergic properties [142]. In fact, Wang and colleagues reported that kaempferol inhibited the iNOS mRNA expression and prostaglandin E2 production in a dose-dependent manner, by inhibiting in part the NF-kB signaling pathway [143]. Furthermore, Park et al. reported that kaempferol was also able to inhibit the activation of inflammatory NF-kB transcription factor via nuclear factor-inducing kinase (NIK)/IkB kinase (IKK) and MAPKs in aged rat kidney [120]. Nonetheless, studies have shown that this flavonoid undergoes excessive first pass metabolism and, as a consequence, displays a bioavailability rate of only 2%, thus making it a good candidate for cutaneous application [142]. Keeping that in mind, Yun Chao and colleagues developed submicron emulsions to be employed as a delivery system for the topical application of kaempferol. These submicron emulsion systems are oil-in-water dispersions with small droplet sizes in the range of 100–600 nm. In comparison with traditional drug delivery vehicles, they are easy to manufacture, are more thermodynamically stable, and exhibit enhanced drug solubilization as well as increased drug permeation rates. Additionally, submicron emulsions were demonstrated to be a potential vehicle for the transdermal and topical delivery of lipophilic and hydrophilic drugs. In this study, kaempferol-loaded submicron emulsions with different water/oil/surfactant/cosurfactant ratios were prepared, and different physiochemical properties (e.g., viscosity, droplet size, permeation rate, lag time, and deposition amount in skin) were determined in order to evaluate the effectiveness of this delivery system for the cutaneous application of kaempferol. Overall, the authors demonstrated that, based on the permeation parameters, including the increase in the cumulative amount of drug over 12 h and deposition in the skin, in addition to a shorter lag time, submicron emulsions may be a promising vehicle for cutaneous application of kaempferol [142].

### 8.2. Examples of Nanocarriers Designed for Other Flavonoid Classes’ Topical Delivery

Isoflavones, are naturally occurring isoflavonoids mainly found in soybeans, soy foods, and legumes. They are non-steroidal compounds that act as phytoestrogens as they exert pseudohormonol activity by binding to estrogen receptors in mammals. The most common isoflavones are genistein and daidzein [5]. Huang and colleagues assessed the potential topical delivery and dermal use of soy isoflavones genistein and daidzein, using α-terpineol and oleic acid as PE, both in vitro and in vivo. As demonstrated in vivo, there was an increase in the uptake of genistein an daidzein, with no toxic effects, and a decrease in the erythema. In vitro studies showed an inhibition of UVB-induced intracellular H_2_O_2_ production and the consequent protection of keratinocytes against UVB radiation, suggesting that a reduction in photodamage to the skin via the topical application of antioxidants could be an efficient way to enrich the endogenous cutaneous protection system [143,144].

Apigenin is a hydrophobic, polyphenolic flavonoid known to possess antioxidant, antimicrobial, anti-inflammatory, antiviral, antidiabetic, and tumor inhibitory activities. In particular, this flavonoid was demonstrated to act as a chemo-preventive by inhibiting the enzyme CYP2C and by preventing the metabolism of many drugs and xenobiotics. Similar to the already mentioned flavonoids, the clinical potential of apigenin is suppressed by its poor aqueous solubility, low oral bioavailability, and rapid metabolism. Thus, the development of novel formulations is a necessary step to overcome these limitations and to improve apigenin delivery [5]. On that matter, several formulations have been developed so far, including liposomes, nanocrystal gel formulations, and self-micro-emulsifying drug delivery systems. Munyendo and colleagues reported that the formulation of D-α-tocopherol acid and polyethylene glycol 1000 succinate (TPGS) stabilized the mixed micelles of apigenin and phospholipids, creating an effective drug delivery vehicle capable of enhancing the bioavailability of this flavonoid [145]. Karthivashan and colleagues prepared “flavonosomes”, which are phytosomes loaded with multiple flavonoids, using phosphatidylcholine as a carrier and evaluated their in vitro pharmacokinetics and toxicity [146]. Shen and co-workers evaluated a novel topical delivery system for apigenin by using soy lecithin-based ethosomes, demonstrating a higher skin targeting capacity and a significant reduction in COX-2 levels in mouse skin inflammation induced by UVB light [147].

Luteolin is another promising flavonoid with potential antiarthritic activity. In addition, due to its lipophilicity, it can be used in topical formulations to treat psoriasis [148]. Niosomes are non-ionic surfactant-based colloidal systems that have the ability to encapsulate both hydrophobic and hydrophilic drugs. Abidin and co-workers prepared luteolin-loaded niosomes using different non-ionic surfactants and characterized them for their in vitro and in vivo antiarthritic activity. The optimized formulation was later converted into gel using Carbopol as a gelling agent for enhanced transdermal luteolin delivery. The in vivo bioactive studies revealed that the niotransgel formulation of luteolin was able to provide good antiarthritic activity, with the results being comparable with standard diclofenac gel formulation [149]. In another study, Shin and colleagues, established a nanoemulsion-based follicular delivery system, in which luteolin was incorporated into oil-in-water nanoemulsions. In vivo studies proved that these luteolin-loaded nanoemulsions possessed hair-growth promotion ability. In fact, when nanoemulsions are formed by the assembly of amphiphilic polymers at the oil/water (O/W) interface, they provide an efficient system for the encapsulation of poorly water-soluble substances, resulting in better bioavailability, accurate dosing, and minimal side effects [150].

Catechins are a group of flavonoids that belong to the flavanol family and are present in high concentrations in a variety of plant-based fruits, vegetables, and beverages. Belonging to this family are catechin, epicatechin (EG), and EGCG. EGCG, in particular, has captured a lot of attention due to its broad spectrum of biological properties, including antioxidant, photoprotective, antiviral, and antibacterial as well as anticancer and neuroprotective effects. Nevertheless, its clinical use has been limited due to its poor systemic absorption and low bioavailability [5]. With the goal to overcome this problem and to increase EGCG clinical applicability, Avadhani and co-workers developed nano-transfersomal formulations of EGCG for an efficient permeation into the SC and delivery into the skin [151]. In addition, hyaluronic acid (HA) was also encapsulated in the transfersomes not only because it is widely distributed in connective tissues and is a main component of the extracellular matrix but also because it is a non-irritating biopolymer and antiaging agent with high biocompatibility, specific viscoelasticity, and hydration and lubrification properties. The optimized transfersomal formulation containing EGCG and HA displayed a high free radical scavenging effect while showing no cell toxicity. In addition, the formulation was able to suppress the MDA and ROS levels to a significant extent in human keratinocytes as well as the expression levels of MMP-2 and MMP-9. The encapsulation of EGCG in the transfersomes resulted in higher skin permeation and deposition of this flavonoid in the skin, compared with plain EGCG. Interestingly, the co-entrapment of HA in the formulation increased both the skin permeation and deposition of EGCG, thus demonstrating that this system constitutes a useful and effective EGCG cutaneous delivery vehicle, with synergistic antiaging and antioxidant benefits [151].

Fang and colleagues assessed the possibility of using multilamellar phosphatidylcholine (PC) liposomes studied for topical and intratumor delivery administration of catechin, EC, and EGCG in nude mice [152,153]. The authors showed that the inclusion of anionic species such as deoxycholic acid and dicetyl phosphate increased the encapsulation of the catechins and the permeability of the lipid bilayers. EGCG performed differently due to its higher lipophilicity. In addition, the authors reported an even higher EGCG encapsulation for deoxycholic acid-liposomes prepared in the presence of 15% ethanol as well as an increased catechin in vitro and in vivo skin permeation and deposition in basal cell carcinomas compared with both the free form and ethanol-free liposomes. This might be attributed to the fact that ethanol-enriched liposomes penetrate easily in the skin due to the increased elasticity conferred by the insertion of alcohol into the PC membranes. The results showed that optimization of the physicochemical features and composition of liposomes could control and improve the delivery of catechins. Moreover, the results suggested that the intratumor administration of liposomes might be an effective approach for the local treatment of solid tumors [152,153].

Overall, there are several strategies that can be adopted to increase the solubility and subsequent bioavailability of flavonoids with therapeutic potential. Although much progress has been recently made, novel drug delivery systems suitable for an optimized topical application should continue to be explored [112,154,155,156,157]. A summary of the therapeutic application of flavonoids and the different nanocarriers used to enhance their delivery to the skin is described in Table 3.

## 9. Concluding Remarks

In the last few years, flavonoids have been extensively studied for their remarkable antioxidant, anti-inflammatory, anticancer, and antibacterial properties. However, their lipophilic nature and poor aqueous solubility invariably lead to limited oral bioavailability. In addition, flavonoids are rapidly degraded and metabolized in the human body, which greatly hinders their clinical application. Thus, oral delivery faces many challenges, and recently, there has been a shift towards the development of new formulations and alternative delivery routes, namely cutaneous administration. Flavonoid encapsulation is also an effective way not only to improve their pharmacokinetics but also to avoid degradation and improve safety. Various novel formulations aiming at cutaneous delivery have been developed with the goal to increase the solubility and permeability of flavonoids across the skin barrier, with minimal adverse effects. However, there is still the need to overcome limitations, such as a sustained release profile and skin retention time, to achieve an effective therapeutic dosage. Within the literature, different experimental protocols have been applied, hampering comparisons and progress to clinical evaluation. There is some indication of a relation between flavonoid’s chemical structure and the most suitable delivery system, but given the diversity of the skin models used, it is not possible to establish such a relation. Other technical issues can limit the translation to industrial process, as laboratorial methods are a challenge to scale up.

Currently, several cutaneous formulations for flavonoids have been described in the literature and some have been patented, which indicates the relevance of these natural compounds, and the difficulty to certify for safety and efficacy towards translation to the market. Stakeholders need to come forward and to support long clinical trials that allow for the evaluation of adverse effects and for the identification of a dosage scheme. Clinical trials must be based on solid preclinical results obtained in appropriate models. The use of animal models (e.g., mice and rabbits) in preclinical studies present limitations related to a lack of similarity to human skin. The research community’s awareness to the search for alternative models finds solutions on mimetic skin models (e.g., reconstituted human epidermis and phospholipid-based permeation assays) as data show a good correlation to human skin absorption and permeability features.

Flavonoids will continue to be explored both as a therapeutic and preventive tool for several disease conditions, alone or in combination (several synergistic effects have been described). A continuous growth in the search for novel strategies to empower flavonoid use is expected given their demonstrated potential as active agent. In the future, limitations on the cutaneous application of flavonoids will be overcome and translational advances towards commercialization will bring novel skin products to the market and to society.

## Figures and Tables

**Table 1:** Main immunological functions of skin cells.

Cell Type	Location in the Skin	Immunological Role	Ref.
Langerhans cells	Epidermis	Sentinel role	[19,25]
		Migration to lymph nodes to induce adaptive immune responses	
		Induction of tolerance	
		Production of pro-inflammatory cytokines and chemokines	
Dermal DCs	Papillary dermis	Antigen presentation	[25]
		Cytokine and chemokine secretion	
Plasmacytoid DCs	Dermis	Production of IFN-α	[21,25]
Macrophages	Papillary and reticular dermis	Antimicrobial activity	[19,25]
		Production of pro- and anti-inflammatory mediators	
		Production of cytokines and chemokines	
		Phagocytosis of pathogenic agents and necrotic debris	
Mast cells	Papillary and reticular dermis	Production of inflammatory mediators involved in allergic responses and asthma	[19]
		Recruitment of immune cells	
		Production of inflammatory cytokines	
B lymphocytes	Reticular dermis	Production of autoantibodies	[27,28]
		specific to components of the skin	
Non-immune cells (keratinocytes and fibroblasts)	Epidermis and reticular dermis	Provide physical barrier and structural integrity	[20,21,22,25]
		Production of inflammatory cytokines and AMPs in response to injury or pathogen invasion	
Neutrophils	Reticular dermis	Phagocytosis during pathogen invasion	[29,30]

		Release of chemo-attractants to recruit other neutrophils to the site of inflammation	
Eosinophils	Reticular dermis	Defense against parasites	[31]

**Table 2:** Synopsis of the main molecular targets and mechanisms of action of flavonoids.

Flavonoid	Molecular Targets	Biological Role	Mechanisms of Action	Ref.
Catechin, Epigallocatechin	ERK, NF-kB, Rac1, AP-1, p38	Anticarcinogenic	Inhibition of iNOS expression	[46,47,48,49]
			Reduction of NF-kB and AP-1 activity	
Apigenin	Akt, ERK, caspase-12, caspase-3, MAPK, ROS, COX-2, IL-6, TNF-α, IL-1β, iNOS, PGE2	Anti-inflammatory, Anticarcinogenic	Inhibition of intercellular adhesion molecule-1 (ICAM-1), VCAM-1, and E-selectin expression	[9,46,47,50,51]
			Inhibition of prostaglandin synthesis and IL-6 production	
Luteolin	Akt, ERK, caspase-12, caspase-3, MAPK, ROS, COX-2, IL-6, TNF-α, IL-1β, iNOS, PGE2	Anti-inflammatory, anticarcinogenic	Inhibition of the upregulation of monocytes adhesion and VCAM-1 expression and NF-kB activity	[9,46,47,50,51]

Quercetin	PKC, AP-1, H_2_O_2_, iNOS, MDA, citrate synthase, MMP-9, MMP-2, COX-2, ERK	Antioxidant, anti-inflammatory	Inhibition of NO production and iNOS protein expression	[46,47,52]
			Inhibition of cyclooxygenase and lipoxygenase activities	
Hesperetin	GSH reductase, iNOS, 3-nitropropionic acid, COX2, NF-kB, IL-1, TNF-α	Antioxidant	Blood lipid-lowering and cholesterol-lowering agents	[46,47,52]

**Table 3:** In vitro and in vivo studies using different nanocarriers for enhanced topical delivery of flavonoids to the skin.

Flavonoid	Nanoformulation	Skin Model	Therapeutic Application	Ref.
Quercetin	Solid lipid nanoparticles	Human skin	Delay UVB radiation-mediated cell damage and necrosis	[139]
	Non-ionic emulsion with high lipid content	Pig ear skin	Inhibition of UVB-induced cutaneous oxidative stress and inflammation	[4]
	Anionic emulsion with low lipid content	Pig ear skin	Inhibition of UVB-induced cutaneous oxidative stress and inflammation	[4]
	Lecithin-chitosan nanoparticles	Male Kunming mice	Topical delivery system with a wide range of applications	[137]
	Lipid microparticles	n.a.	Enhance quercetin stability in topical formulations	[136]
	Colloidal silica emulsion	Human skin	Optimization of a formulation with enhance penetration into human SC	[156]
	Chitosan nanoparticles	HaCaT cells	Potential therapeutic agent for topical use against UVB radiation	[138]
	Penetration Enhancer containing Vesicles (PEVs)	New born pig skin	New formulation for dermal delivery of quercetin, with various therapeutic applications	[140]
	Polylactide nanocapsules; Multilamellar liposomes; Niosomes	Subcutaneous injection in amistogote-infected hamsters	Antileishmanial agent	[3,157]
	Liposomes with penetration enhancing vesicles (PEV)	Female CD-1 mice	Anti-inflammatory agent	[5,157]
	Lipid nanocapsules	Acute monocytic leukemia cell line (THP1–1 cell)	Antioxidant, anti-inflammatory agent	[5,158]
	Nanoparticle suspension	Mice	Antioxidant agent	[5,149]
Catechins	Multilamellar phosphatidylcholine-liposomes	Female nude mouse (Balb/c-nu, 6–8 weeks old)	Use of liposomes for the local delivery, including skin and tumor deposition, of polyphenols	[3,151]
	Ethanol enriched liposomes	Female nude mouse (Balb/c-nu, 6–8 week)	Antioxidant and chemopreventive activity	[152]
	Cream	Iranian rabbits	Wound healing effect	[5,159]
	Tansfersomes containing EGCG and hyaluronic acid (HA)	HaCaT cells	Synergize the UV radiation-protective ability of EGCG and HA along with imparting antioxidant and antiaging effects	[5,150]
Genistein	Nanoemulsion	Pig ear skin	New formulation for dermal delivery of genistein, with various therapeutic applications	[3,160]
Kaempferol	Submicron emulsions	Sprague Dawley rat	Promising vehicle for topical kaempferol application	[142]
Resveratrol	Solid lipid Nanoparticles	Porcine skin	Protection from photodegradation	[161]
Resveratrol + curcumin	Lipid-core Nanocapsules	Human skin	Increase skin delivery of resveratrol	[162]
	Niosomes	Cell rabbit skin	Increase skin delivery of resveratrol Increased antioxidant activity	[163]
Hesperetin, hesperidin	Microemulsion	Guinea pigs	Whitening effect	[5,164]
	Topical matrix film	Albino rabbits	Release of hesperetin in posterior of eye	[5,165]
	Microemulsion based ointment	Wistar rats	Skin irritation	[5,166]
Naringenin	Gel	HRS/J mice	Antioxidant and anti-inflammatory agent	[5,162]
	Nanoparticles	Wistar rats	Photoprotective, antioxidant agent	[5,162]
Apigenin	Phospholipid phytosomes	Albino rats	Antioxidant agent	[5,167]
	Ethosomes	Konmin mice	Anti-inflammatory agent	[5,168]
Anthocyanin	Niosome gel	Male Wistar rats	Anti-inflammatory agent	[5,169]
Luteolin	Luteolin in olive oil	ICR mice	Anti-inflammatory agent	[5,170]
	Luteolin-loaded niosomes/Niosomal transgel	Albino Wistar rats	Treatment of arthritis	[148]
	Nanoemulsion	C57BL/6 mice	Growth promoting effect	[149]
